# Alcohol use among HIV-positive women of childbearing age, United States, 2013–2014

**DOI:** 10.1080/09540121.2020.1808161

**Published:** 2020-08-18

**Authors:** Emma L. Frazier, Marissa B. Esser, Lela R. McKnight-Eily, Wen Zhou, Pollyanna R. Chavez

**Affiliations:** a Division of HIV/AIDS Prevention, National Centers for HIV, Viral Hepatitis, STD and TB Prevention, Centers for Disease Control and Prevention; b Excessive Alcohol Use Prevention Team, National Center for Chronic Disease and Prevention and Health Promotion, Centers for Disease and Prevention; c Prenatal Alcohol, Opioid, and Substance Exposure Team, National Center on Birth Defects and Developmental Disabilities; d ICF International, Atlanta

**Keywords:** HIV, women, alcohol consumption, binge drinking, pregnancy

## Abstract

More than one-quarter of the adults living with diagnosed HIV infection in the US are women. Binge drinking (i.e., ≥4 alcoholic drinks per occasion for women) is associated with poor HIV treatment compliance, HIV incidence, and unplanned pregnancy. However, little is known about the prevalence of binge drinking among women of childbearing age who are living with HIV (WLWH) and health risk behaviours among those who binge drink. Using the 2013–2014 data cycles of Medical Monitoring Project, we assessed the weighted prevalence of drinking patterns by socio-demographic, clinical and reproductive characteristics of 946 WLWH. Logistic regression was used to calculate unadjusted and adjusted prevalence ratios and 95% confidence intervals. Overall, 39% of WLWH reported current drinking and 10% reported binge drinking. Compared to non-drinkers, binge drinkers were less likely to adhere to antiretroviral therapy (ART) or be virally suppressed. In multivariate analyses, binge drinking among WLWH was associated with smoking, drug use, and reduced ART adherence compared to non-drinkers, increasing the likelihood of negative clinical outcomes. WLWH may benefit from a comprehensive approach to reducing binge drinking including alcohol screening and brief interventions and evidence-based policy strategies that could potentially improve adherence to HIV treatment.

## Introduction

In 2016, nearly 1 million persons in the US were living with diagnosed HIV infection (Centers for Disease Control and Prevention (CDC), 2019). Women account for more than one in four of adults living with diagnosed HIV infection in the US (CDC, 2017). Additionally, more than three in five women living with HIV (WLWH) were diagnosed prior to age 45 (CDC, 2017).

Any alcohol use is associated with increased all-cause mortality in the general population (Griswold et al., 2018; Wood et al., 2018). Excessive alcohol use, including binge drinking, is particularly concerning among WLWH because it has been associated with risky sexual behaviour (Scott-Sheldon et al., 2016; Shuper et al., 2010; Stein et al., 2005), increased risk for all-cause mortality (Neblett et al., 2011), reduced efficacy of and poor health outcomes related to HIV treatment (Monroe et al., 2016; Vagenas et al., 2015), and reduced adherence to antiretroviral medications and viral suppression (Barai et al., 2017; Vagenas et al., 2015). Binge drinking among WLWH also increases the risk of dangerous interactions between alcohol and antiretroviral medications, such as the development of liver disease (Barve et al., 2010).

Advances in HIV treatment have made it possible for many WLWH to consider having a pregnancy without concerns of vertical transmission (Blair et al., 2004; Haddad et al., 2017). Data from the 2007–2008 Medical Monitoring Project (MMP) showed that one-quarter of WLWH of childbearing age had one or more pregnancies after being diagnosed with HIV. Most (85%) of these pregnancies were unplanned, in contrast to 45% of pregnancies among women in the general population that were unplanned (Finer & Zolna, 2014; Sutton et al., 2014). Binge drinking has been associated with increased risk of unplanned pregnancies among women in the general population (Naimi et al., 2003); however, to the authors’ knowledge, the association between binge drinking and reproductive outcomes has not been examined among WLWH.

The primary objectives of this study, therefore, are to estimate the prevalence of current drinking and binge drinking among WLWH who are of childbearing age and receiving medical care, and to assess how drinking patterns vary in this population, with a focus on binge drinking, based on socio-demographic characteristics, health risk behaviours, clinical characteristics (e.g., Stage of HIV infection), and reproductive outcomes (e.g., Pregnancy history). The study findings can inform the development and implementation of a comprehensive strategy to effectively prevent excessive drinking and related harms among WLWH.

## Methods

We analyzed matched interview and medical record data from the combined 2013–2014 data collection cycles of the Medical Monitoring Project (MMP), an HIV surveillance system designed to produce annual, nationally representative estimates of the characteristics of HIV-positive adults receiving medical care in the US. The MMP methods, including non-response bias analysis and weighting techniques, have been described in detail elsewhere (Iachan et al., 2016).

In brief, the 2013–2014 MMP cycles used a three-stage, probability-proportional-to-size sampling method. First, selected and funded US states and one territory were sampled. Second, eligible facilities providing outpatient HIV care in those geographic areas were sampled. Third, HIV-positive patients meeting specific eligibility criteria were then sampled in each of the selected outpatient facility locations. To be eligible for this study, patients had to have received medical care in participating facilities between January and April in the cycle year for which they were sampled. Data from MMP were weighted to produce estimates that represent HIV-positive patients aged 18 or older receiving care in the US.

A total of 23 health jurisdictions (California, Chicago (IL), Delaware, Florida, Georgia, Houston (TX), Illinois, Indiana, Los Angeles County (CA), Michigan, Mississippi, New Jersey, New York state, New York City (NY), North Carolina, Oregon, Pennsylvania, Philadelphia (PA), Puerto Rico, San Francisco (CA), Texas, Virginia, and Washington state) were funded, and conducted data collection for both the 2013 and 2014 cycles of MMP. Trained interviewers and medical record abstractors collected data in the two data collection cycles from June 2013 through May 2014 (2013 data cycle) and June 2014 through May 2015 (2014 data cycle). After adjusting for eligibility, the average facility response rate was 85.7%, and the average patient response rate was 55.3%.

### Analytic sample

We combined MMP 2013–2014 data cycles (n=10,184). The study population was then restricted to WLWH, aged 18–44 years. Of the 10,184 participating patients, 2,766 (27%) were WLWH. Of the 2,766 WLWH, 957 (35%) were aged 18–44 years. Of these 957 WLWH, 11 (1%) were excluded because they did not respond to questions on their drinking status, resulting in a final analytic sample of 946 women.

### Alcohol consumption measures

We assessed alcohol consumption using the following three questions: “During the past 12 months, how often did you drink alcohol?” [Daily, weekly, monthly, less than monthly, never]. Those who reported drinking any alcohol were asked: “During the past 30 days, on how many days did you have an alcoholic drink?” And “During the past 30 days, on how many days did you have 4 or more alcoholic drinks in one sitting?”. A standard drink of alcohol contains 14 grams of pure ethanol (US Department of Health and Human Services and US Department of Agriculture, 2015). We created three mutually exclusive drinking categories, consistent with other studies that have focussed on risks associated with binge drinking using national surveys (Esser et al., 2019). Non-drinkers were defined as women who did not report drinking any alcohol in the past 30 days. Current drinkers who did not binge drink (referred to as “current/non-binge”) were defined as women who reported consuming ≥1 drink in the past 30 days and did not report consuming ≥4 drinks in a sitting. Binge drinkers were defined as women who reported consuming ≥4 drinks during at least one sitting in the past 30 days.

### Other measures

We analyzed drinking patterns of respondents by age at interview, race/ethnicity, highest educational attainment, household income (at or below poverty level), length of time since HIV diagnosis, type of health insurance coverage, reproductive health outcomes, health risk behaviours, and use of non-HIV medical services. Poverty status was based on the US Department of Health and Human Services poverty guidelines for the calendar year for which a patient’s combined household income was assessed. Reproductive health outcomes included number of times pregnant, number of unplanned pregnancies, and births since first HIV diagnosis (reproductive outcome questions are available in Appendix Table 1). Health risk behaviours included current smoking, any drug use for non-medical purposes, and any condomless sex with at least one male partner who was HIV-negative or of unknown status in the past 12 months. Women also answered questions about the need for non-HIV medical services, including drug or alcohol counselling and treatment services in the 12 months prior to the interview (unmet needs questions are available in Appendix Table 1).

We analyzed drinking patterns by clinical outcomes, including HIV disease stage, viral suppression, and adherence to antiretroviral therapy (ART). We categorised disease stage based on information abstracted from the participant’s medical record using the following CDC HIV surveillance case definitions (Schneider et al., 2008): Stage 1, no AIDS and nadir CD4 count ≥ 500 cells/μL (or CD4% ≥ 29); Stage 2, no AIDS and nadir CD4 count 200–499 cells/μL (or CD4% 14-<29); Stage 3 (AIDS) or nadir CD4 count 0–199 cells/μL (or CD4% <14). We defined viral suppression as the most recent viral load in the past 12 months <200 copies/ml or undetectable. We assessed self-reported ART adherence for the three days prior to the patient interview, using AIDS Clinical Trials Group measures (Chesney et al., 2000). Specifically, a patient was considered adherent if they were currently taking ART and had taken all prescribed doses of the ART (i.e., Pills/spoonfuls/injections of ART medications) during the previous three-day period. If both conditions did not apply, a woman was considered non-ART adherent.

### Statistical analyses

We computed weighted prevalence estimates and 95% confidence intervals (CIs) to describe the socio-demo-graphic and clinical characteristics, and health risk behaviours of women in our study. The Rao Scott chi-square test was used to assess significant differences in the prevalence of drinking patterns by selected characteristics.

We assessed the prevalence of each drinking pattern for each selected characteristic. We performed logistic regression analyses to compute unadjusted prevalence ratios (PR) and 95% CIs of the prevalence of each drinking pattern within selected characteristics. Lastly, two multivariable logistic regression models were used to calculate adjusted prevalence ratios (aPRs), based on predicted marginals, and 95% CIs for correlates of both current/non-binge and binge drinking compared to non-drinkers in the past 30 days. Factors associated with either current/non-binge or binge drinking at *p*<0.10 and/or a priori evidence based on associations from the literature (e.g., Age) were used for initial inclusion in the multivariable regression models. Significantly associated factors, including a priori variables, were included in the final model (*p*<0.05).

All analyses were performed using SAS 9.4 (SAS Institute, Cary, NC) and SAS-callable SUDAAN 10.0.1 (RTI International, Research Triangle Park, NC) and clustering, unequal selection probabilities, and non-response were accounted for by weighting.

### Ethics statement

Consistent with guidelines for defining public health research, CDC considers MMP to be public health surveillance used for disease control, programme, or policy purposes. Local institutional review board approval was obtained from participating states, territories, and facilities when required. Informed consent was obtained from all interviewed participants.

## Results

The majority of WLWH of childbearing age were aged 30 years or older (80.7%), and of non-Hispanic, black race (61.6%) (Table 1). Most of the women had a high school education or less (60.8%); lived at or below the federal poverty level (70.2%); had public health insurance only (62.5%) and had been diagnosed with stage 3 (AIDS) (57.9%). Nearly 1 in 3 (29.0%) of the women were current/non-binge drinkers, and 1 in 10 (10.4%) were binge drinkers. In addition to engaging in binge drinking, binge drinkers also reported drinking more frequently and consuming a greater total number of alcoholic drinks during the past 30 days, compared with current/non-binge drinkers (Appendix Table 2), further validating how binge drinkers’ alcohol consumption patterns differ from current/non-binge drinkers.

Among women with at least one unplanned pregnancy since their HIV diagnosis, 30.7% (CI: 25.3-36.1) were current/non-binge drinkers and another 11.1% (CI: 7.7-14.6) were binge drinkers (Table 1). Among women who engaged in condomless sex with at least one partner who was HIV-negative or of unknown HIV status in the past 12 months, 35.3% were current/non-binge drinkers and another 15.4% were binge drinkers.

**Figure 1.**
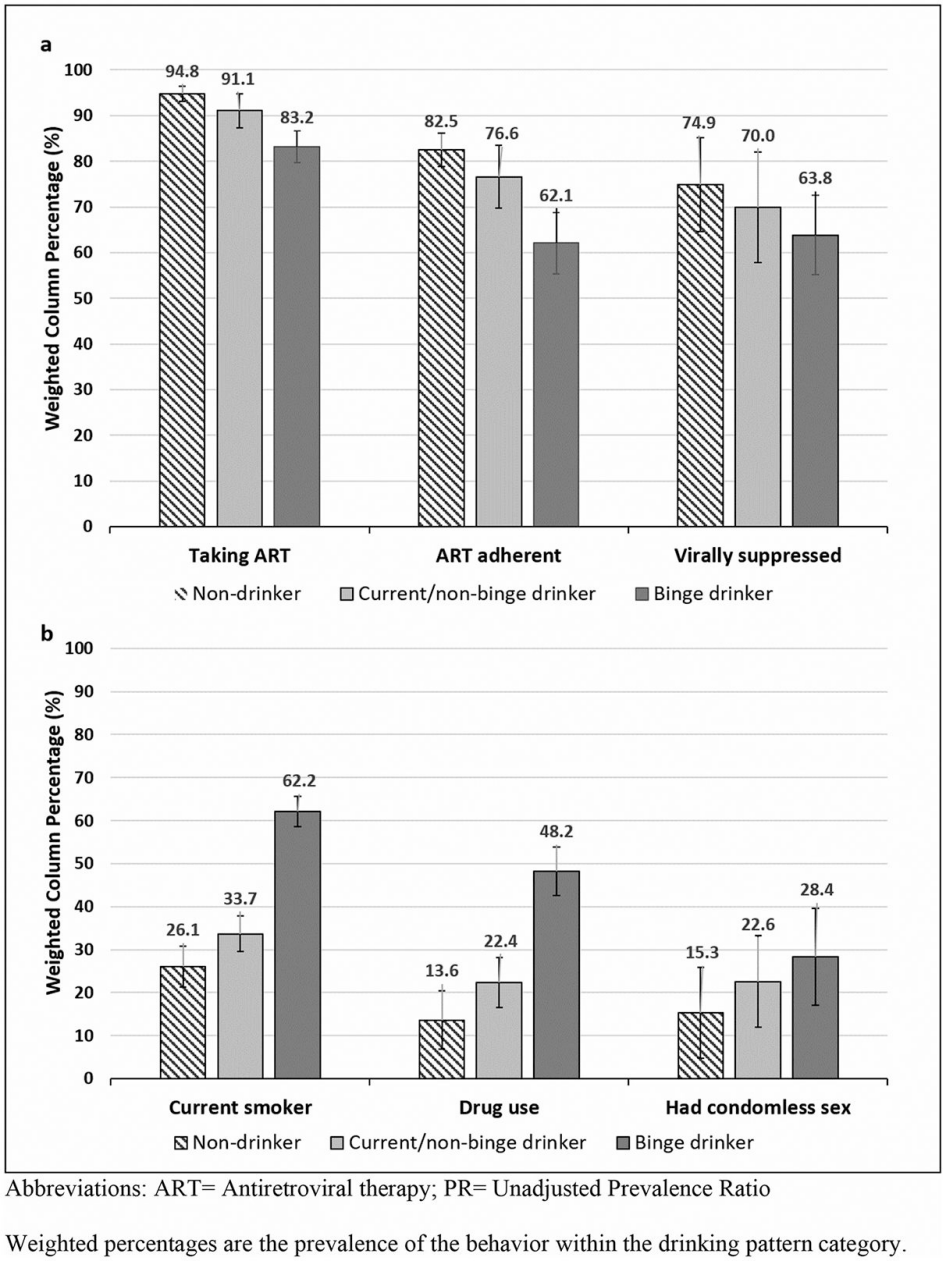
a. Prevalence of Clinical Characteristics among HIV-positive Women of Childbearing Age in Care, Stratified by Drinking Status – Medical Monitoring Project, 2013–2014. b. Prevalence of Health Risk Behaviors among HIV-positive Women of Childbearing Age in Care, Stratified by Drinking Status – Medical Monitoring Project, 2013–2014.Abbreviations: ART = Antiretroviral therapy; PR = Unadjusted Prevalence Ratio.Weighted percentages are the prevalence of the behaviour within the drinking pattern category.Time period: In the past 12 months, unless otherwise noted. All measures are self-reported unless otherwise noted.Non-drinkers were defined as women who did not report drinking any alcohol in the past 30 days.Current/non-binge drinkers were defined as women who reported consuming ≥1 drink in the past 30 days and did not report consuming ≥4 drinks in a sitting.Binge drinkers were defined as women who reported consuming ≥4 drinks during at least one sitting in the past 30 days.Drug use includes non-injection and injection use for non-medical reasons.ART adherent was defined as patients who self-report that they are currently taking ART and were 100% dose adherent in the past 3 days. A patient is defined as 100% adherent if they took their ART doses or set of pills/spoonfuls/injections of ART medications as prescribed by a health care provider in the last 3 days. Otherwise, they were considered as not adherent.Recent viral suppression is defined as the most recent viral load in the past 12 months prior to the interview as undetectable or <200 copies/ml. This information is based on data as recorded by medical record abstraction during the past 12 months prior to interview.

When assessed within each drinking pattern, binge drinkers had less favourable clinical characteristics and more health risk behaviours. Compared to non-drinkers, WLWH who binge drank were significantly less likely to be taking ART (83.2% (CI: 79.2-93.5) vs. 94.8% (CI: 93.0-96.5)), adhere to ART (62.1% (CI: 50.0-74.2) vs. 82.5% (CI: 78.8-86.2)), and be virally suppressed (63.8% (CI: 55.1-72.5) vs. 74.9%, (CI: 71.3-78.4)) (Figure 1a). Behaviourally, compared to non-drinkers, binge drinkers were significantly more likely to be a current smoker (62.2% (CI: 51.6-72.8) vs. 26.1% (CI: 21.2-30.9)) and use drugs for non-medical reasons (48.2% (CI: 37.5-58.8) vs. 13.6% (CI: 9.5-17.7)) (Figure 1b). WLWH who binge drank were also more likely to report condomless sex with at least one male partner in the past year than non-drinkers (28.4% (CI: 17.1-39.7) vs. 15.3% (CI: 11.9-18.8)).

Women aged 18–29 years (aPR=1.45, CI: 1.03-2.02) and 30–39 years (aPR=1.27, CI: 1.03-1.57) were more likely to be current/non-binge drinkers than non-drinkers, compared to women aged 40–44 years, even after controlling for potential confounding factors (Table 2). WLWH with more than a high school education were also significantly more likely to be current/non-binge drinkers compared to those with less than a high school education (aPR=1.39, CI: 1.04-1.86). WLWH who lived above the poverty level (aPR=1.47, CI: 1.18-1.83); used injection or non-injection drugs (aPR=1.31, CI: 1.04-1.65); and needed drug or alcohol counselling and treatment services (aPR=1.73, CI: 0.97-3.09) were more likely to be current/non-binge drinkers than non-drinkers. In addition, women who smoked were significantly more likely to binge drink that to be non-drinkers (aPR=2.31, CI: 1.38-3.87); as were women who used injection or non-injection drugs (aPR=2.25, CI: 1.44-3.52); and those were non-ART adherent (aPR=1.56, CI: 1.04-2.35).

## Discussion

This study found that nearly 1 in 3 WLWH of childbearing age who were receiving medical care were current/non-binge drinkers, and 1 in 10 were binge drinkers during the past 30 days. WLWH who smoked or used drugs were over twice as likely to binge drink than not drink at all, and fail to adhere to ART, substantially increasing their risk of adverse health outcomes, including the progression of HIV disease (Schneider et al., 2012). Among WLWH who binge drink, more than 25% reported engaging in condomless sex with at least one male partner in the past 12 months, compared to about 15% of non-drinkers.

The finding that 10% of WLHW reported binge drinking is notable due to the potential consequences for this population, although it is lower than among women in the general population (17.6%) (CDC, 2016). However, the 39.4% of women in this study who consumed alcohol is consistent with other studies that have documented that 29-45% of WLWH consume alcohol (Chander et al., 2006; Cook et al., 2009; Galvan et al., 2002; Neblett et al., 2011). Given the unique medical considerations for this population, even the consumption of alcohol at levels below the binge drinking threshold may still put WLWH at risk for experiencing health complications (Shuper et al., 2010). For example, consuming alcohol while using ART could increase the risk of liver damage (Schneider et al., 2012). Future research could assess how health risk behaviours, clinical characteristics, and reproductive outcomes vary using other measures of alcohol use (e.g., Frequency of drinking days, frequency of binge drinking).

The finding that WLWH who smoke and those who used drugs are twice as likely to binge drink corroborates other research that has documented the co-use of alcohol, tobacco, and other drugs among HIV-infected persons (Silverberg et al., 2018). The independent association between decreased ART adherence and binge drinking is also consistent with existing research suggesting the potential for negative health outcomes among this population (Barai et al., 2017; Baum et al., 2010; Chander et al., 2006; Cook et al., 2009; Hendershot et al., 2009; Williams et al., 2016). Though this study was not able to assess why women who did not adhere to ART were more likely to binge drink, other studies have found that HIV-positive patients may intentionally miss doses because they believe alcohol consumption can increase the toxicity of ART medications (Kalichman et al., 2013; Pellowski et al., 2016).

In this study, more than 2 in 5 WLWH who had ≥1 unplanned pregnancy since their HIV diagnosis were drinkers, suggesting a potential for alcohol-exposed pregnancies in this population. Although WLWH in the US rarely transmit HIV to their children (Sutton et al., 2014), alcohol-exposed pregnancies can lead to other adverse outcomes, including premature birth and fetal alcohol spectrum disorders (FASDs) (Green et al., 2016). Given these study findings that WLWH who binge drink had increased rates of engaging in clinical and health risk behaviours, such as being less likely to take ART, adhere to ART, be virally suppressed, and more likely to report condomless sex with a male partner of negative or unknown HIV status, a comprehensive, multi-faceted approach may help prevent binge drinking and related harms among WLWH. This includes alcohol screening and brief intervention (alcohol SBI), which has been recommended by the US Preventive Services Task Force for adults in primary care (18 years and older), including pregnant women, since 2004 (US Preventive Services Task Force, 2018). While the assessment of alcohol use has been a lower priority in the myriad of care needs for HIV-positive persons (Chander et al., 2006), it has been suggested for persons with HIV (Strauss et al., 2012). Some successful efforts have been made to incorporate alcohol SBI in Ryan White HIV clinics (Graham et al., 2016). CDC has developed guidelines to help primary care practices implement alcohol SBI in a routine manner (CDC, 2014). Essential components of this strategy include using a validated instrument to identify persons who drink excessively, followed by a brief intervention for those who do, and a referral to treatment for those identified as having a severe alcohol use disorder (CDC, 2014). The key elements of alcohol SBI can also be effectively delivered electronically using computers or mobile devices. This approach, known as electronic SBI (e-SBI), which has been recommended by the Community Preventive Services Task Force (Tansil et al., 2016), can be administered in a variety of settings, including HIV clinics. The implementation of alcohol SBI, either in person or using e-SBI, may be beneficial for reducing excessive alcohol use among WLWH.

The implementation of evidence-based policy strategies, including those recommended by the Community Preventive Services Task Force, could also be considered as part of a comprehensive approach for reducing binge drinking among WLWH. Such interventions include increasing alcohol taxes (Elder et al., 2010), regulating the density of alcohol outlets (Campbell et al., 2009), and having commercial host liability laws that hold retail establishments liable for injuries or harms caused by illegal service to intoxicated or underage patrons (Rammohan et al., 2011). Evidence suggests that increasing alcohol taxes could effectively reduce sexually transmitted infections. In Illinois, an alcohol tax increase was associated with a reduction in state-wide rates of gonorrhea by 21% and of chlamydia by 11%, and these reductions were greater among non-Hispanic black than among other racial and ethnic groups (Staras et al., 2014).

### Limitations

The current study is subject to some limitations. First, data were limited to WLWH of childbearing age receiving HIV care; women who were not in care or undiagnosed were excluded. It is possible that women in care may be less likely to drink or drink excessively than those who are unaware of their HIV infection or who are not receiving treatment. Thus, our findings cannot be generalised to all WLWH of childbearing age. Second, several factors, including smoking, sexual behaviour, and self-reported alcohol and drug use may be underestimated due to social desirability and recall biases. Third, there was a small number of women in some sub-groups, such as women who had not had an unplanned pregnancy who binge drank, which affected our ability to assess some statistically significant differences. Fourth, with the modest MMP response rates, nonresponse bias is possible. Our probabilistic sampling frame allowed us to examine characteristics of sampled patients (e.g., Sex, age, race, length of time since HIV diagnosis) to conduct a comparative analysis of respondents and non-respondents. Research has shown that well-con-structed samples with moderate response rates have a reduced risk of bias (Groves & Peytcheva, 2008). Fourth, given the cross-sectional nature of the MMP, causality cannot be inferred about the relationship between HIV, alcohol use, and health risk behaviours.

## Conclusions

WLWH of childbearing age who engaged in other health risk behaviours (e.g., Drug use) were twice as likely to binge drink, increasing the likelihood of negative clinical outcomes. The adoption of a comprehensive approach, including widespread use of evidence-based alcohol policy strategies and the implementation of alcohol SBI with referral to specialised treatment for those who need it, could help reduce excessive drinking among WLWH (Campbell et al., 2009; Elder et al., 2010; Rammohan et al., 2011). Reducing excessive drinking among WLWH might, in turn, improve ART adherence.

## Supplementary Material

Sup Table 2

Sup Table 1

## Figures and Tables

**Table 1.** Selected Characteristics of HIV-positive Women of Childbearing Age (18-44 years) in Care by Drinking Status – Medical Monitoring Project, 2013–2014.

	Total		Non-drinking^a^		Current/non-binge drinking^b^		Binge drinking^c^		
Characteristics	n	%^d^	n	% (95% CI)	n	% (95% CI)	n	% (95% CI)	*p*-value^e^
**Total**	946		574	60.6 (57.3-63.9)	272	29.0 (26.4-31.6)	100	10.4 (8.4-12.4)	
**Age at interview, in years**									0.14
18–29	172	19.3 (16.3-22.3)	95	55.1 (45.4-64.7)	56	32.2 (23.1-41.3)	21	12.7 (8.6-16.8)	
30–39	409	44.2 (40.6-47.9)	250	60.1 (55.1-65.2)	124	31.4 (26.6-36.2)	35	8.4 (6.1-10.7)	
40–44	365	36.5 (32.3-40.7)	229	64.1 (58.8-69.5)	92	24.4 (20.8-28.0)	44	11.5 (7.1-15.9)	
**Race/ethnicity**									0.12
Non-Hispanic black	595	61.6 (53.5-69.6)	361	61.1 (57.0-65.1)	179	30.4 (27.0-33.8)	55	8.5 (6.5-10.5)	
Hispanic or Latino^f^	179	18.8 (10.7-26.8)	111	61.8 (52.4-71.1)	48	27.7 (18.1-37.3)	20	10.5 (5.1-15.9)	
Non-Hispanic white	135	15.6 (11.8-19.4)	81	59.3 (49.3-69.4)	37	27.3 (19.5-35.2)	17	13.3 (5.8-20.9)	
Other^g^	37	4.1 (2.0-6.2)	21	53.6 (38.2-69.0)	8	— h	8	— h	
**Highest educational attainment**									**0.02**
<High school	257	27.2 (24.3-30.1)	172	67.5 (60.9-74.2)	56	22.7 (18.3-27.1)	29	9.8 (4.7-14.9)	
High school graduate or GED	307	33.6 (29.4-37.8)	190	60.7 (52.1-69.4)	77	26.0 (20.9-31.0)	40	13.3 (7.9-18.7)	
>High school	381	39.2 (34.8-43.5)	212	55.8 (51.3-60.3)	138	36.0 (31.0-40.9)	31	8.3 (5.7-10.8)	
**Living at or below federal poverty level** ^i^									**<0.01**
No	267	29.8 (25.6-34.0)	135	48.6 (42.0-55.1)	107	41.8 (35.4-48.2)	25	9.6 (6.3-12.9)	
Yes	625	70.2 (66.0-74.4)	403	65.2 (60.5-69.9)	153	24.2 (20.3-28.1)	69	10.6 (7.3-13.8)	
**Health insurance coverage**									**<0.01**
Any private insurance	181	19.3 (15.6-23.0)	95	51.2 (44.3-58.1)	72	41.6 (35.1-48.1)	14	7.2 (3.7-10.8)	
Any public insurance^j^	594	62.5 (56.5-68.4)	369	62.3 (57.5-67.2)	154	25.6 (21.8-29.5)	71	12.0 (9.2-14.9)	
Ryan White coverage only or Uninsured	171	18.2 (13.5-22.9)	110	64.8 (58.0-71.5)	46	27.2 (20.6-33.9)	15	8.0 (4.5-11.5)	
**Experienced homelessness** ^k^									0.30
No	859	90.8 (88.1-93.5)	522	60.8 (57.3-64.3)	251	29.3 (26.5-32.1)	86	9.9 (8.0-11.8)	
Yes	87	9.2 (6.5-11.9)	52	59.1 (49.3-68.9)	21	26.0 (16.4-35.5)	14	14.9 (7.4-22.4)	
**Pregnancy since HIV diagnosis**									0.70
No	524	56.2 (53.0-59.5)	323	61.4 (57.7-65.2)	144	27.9 (24.3-31.5)	57	10.6 (8.1-13.2)	
Yes	421	43.8 (40.5-47.0)	250	59.5 (53.8-65.1)	128	30.5 (26.1-34.8)	43	10.1 (6.9-13.2)	
**Unplanned pregnancy** ^l^									0.30
No	95	21.7 (17.5-25.8)	61	63.8 (55.8-71.9)	28	29.9 (22.7-37.2)	6	h	
Yes	325	78.3 (74.2-82.5)	188	58.1 (51.6-64.6)	100	30.7 (25.3-36.1)	37	11.1 (7.7-14.6)	
**Number of live births** ^l^									0.33
None	75	17.9 (14.3-21.5)	42	54.4 (42.9-66.0)	22	31.3 (20.9-41.7)	11	14.3 (6.2-22.4)	
≥1	345	82.1 (78.5-85.7)	207	60.4 (54.5-66.4)	106	30.4 (25.3-35.4)	32	9.2 (6.3-12.0)	
**Current smoker**									**<0.01**
No	647	68.0 (63.6-72.3)	426	66.0 (62.7-69.3)	183	28.2 (25.1-31.3)	38	5.8 (4.1-7.4)	
Yes	298	32.0 (27.7-36.4)	148	49.4 (42.2-56.5)	88	30.5 (24.6-36.3)	62	20.2 (14.9-25.4)	
**Any drug use** ^m^									<**0.01**
No	760	80.3 (75.7-84.8)	498	65.3 (61.9-68.7)	208	28.0 (24.8-31.2)	54	6.7 (5.1-8.3)	
Yes	186	19.7 (15.2-24.3)	76	41.7 (35.6-47.9)	64	33.0 (26.9-39.1)	46	25.3 (19.6-31.0)	
**Had any condomless sex with at least one negative or unknown male partner**									**<0.01**
No	736	81.2 (77.9-84.5)	464	63.1 (59.4-66.8)	202	27.9 (24.8-31.1)	70	9.0 (7.0-11.0)	
Yes	178	18.8 (15.5-22.1)	89	49.3 (42.1-56.5)	63	35.3 (27.5-43.1)	26	15.4 (9.0-21.8)	
**Needed drug or alcohol counselling or treatment services** ^n^									**<0.01**
No	868	92.2 (89.3-95.1)	529	60.9 (57.9-64.0)	259	30.2 (27.4-33.1)	80	8.8 (7.0-10.7)	
Yes	77	7.8 (4.9-10.7)	45	57.3 (43.2-71.3)	12	14.3 (7.9-20.7)	20	28.5 (16.4-40.5)	
**HIV disease stage** ^o,p^									0.40
Stage 1 (HIV)	120	13.1 (10.6-15.5)	65	53.1 (38.7-67.4)	39	32.5 (22.0-43.0)	16	14.4 (6.7-22.2)	
Stage 2 (HIV)	275	29.0 (25.5-32.5)	172	62.3 (57.2-67.3)	73	26.8 (21.9-31.7)	30	10.9 (7.4-14.4)	
Stage 3 (AIDS)	549	57.9 (54.0-61.9)	336	61.5 (57.7-65.4)	159	29.3 (25.8-32.8)	54	9.2 (7.0-11.4)	
**ART adherent** ^q^									**<0.01**
No	196	21.4 (17.6-25.2)	100	49.3 (40.3-58.3)	63	31.8 (24.6-39.1)	33	18.8 (12.9-24.8)	
Yes	726	78.6 (74.8-82.4)	458	63.3 (59.9-66.7)	201	28.3 (25.1-31.6)	67	8.4 (6.2-10.6)	
**Virally suppressed** ^o,r^									**0.04**
No	263	27.7 (24.2-31.2)	144	55.0 (49.9-60.1)	83	31.4 (26.6-36.3)	36	13.6 (8.7-18.4)	
Yes	683	72.3 (68.8-75.8)	430	62.8 (59.0-66.6)	189	28.1 (24.7-31.5)	64	9.2 (7.3-11.0)	

Abbreviations: n = unweighted sample size; ART = Antiretroviral therapy; CI = Confidence interval; GED = General Education Development **Bold** indicates significance at *p*<0.05.

Time period: In the past 12 months, unless otherwise noted. All measures are self-reported unless otherwise noted.

a Defined as women who did not report drinking any alcohol in the past 30 days.

b Defined as women who reported consuming ≥1 drink in the past 30 days and did not report consuming ≥4 drinks in a sitting.

c Defined as women who reported consuming ≥4 drinks during at least one sitting in the past 30 days.

d Percentages are weighted column percentages.

e Chi-square *p*-value is based on the Rao-Scott chi-square.

f Hispanics or Latinos can be of any race.

g Other includes multi-racial groups, other racial groups, and missing racial groups.

h Estimates are considered to be unstable and are not reported when the coefficient of variation >0.30 and n<10.

i The number and percentage of participants meeting current poverty guidelines were determined using the U.S. Department of Health and Human Services poverty guidelines that corresponded to the calendar year about which the combined household income was asked.

j Public insurance includes Medicare, Medicare, Medicare or Medicaid with Ryan White, or any local or nationally funded health insurance.

k Homelessness was defined as having lived on the street, in a shelter, in a single room occupancy hotel, or in a car in the past 12 months.

l Among all women who reported having had ≥1 pregnancy since first HIV diagnosis.

m Drug use includes non-injection and injection use for non-medical reasons.

n Needed drug or alcohol counselling or treatment services is defined as patients who responded affirmatively that they needed drug or alcohol counselling or treatment services in the past 12 months. Among those who responded affirmatively that they needed the services (n=77), 66/77 (85.7%) responded that they needed and received drug or alcohol counselling or treatment services in the past 12 months; 11/77 (11.3%) responded that they needed and did not receive drug or alcohol counselling or treatment services in the past 12 months.

o Based on medical record abstraction data in the past 12 months prior to interview.

p The stages are defined as follows: Stage 1 (HIV) – no AIDS and nadir CD4>=500 copies/μL (or CD4% >=29); Stage 2 (HIV) – no AIDS and nadir CD4 count between 200–499 copies/μL (or CD4% 14-<29); and Stage 3 (AIDS) – AIDS or nadir CD4 between 0–199 copies/μL (or CD4% <14%).

q ART adherent was defined as patients who self-report that they are currently taking ART and were 100% dose adherent in the past 3 days. A patient is defined as 100% adherent if they took their ART doses or set of pills/spoonfuls/injections of ART medications as prescribed by a health care provider in the last 3 days. Otherwise, they were considered as not adherent.

r Recent viral suppression is defined as the most recent viral load in the past 12 months prior to the interview as undetectable or <200 copies/ml. This information is based on data as recorded by medical record abstraction.

**Table 2.** Crude and Adjusted Prevalence Ratios for Selected Characteristics of HIV-positive Women of Childbearing Age (18–44 years) in Care by Drinking Status – Medical Monitoring Project, 2013–2014.

	Current/non-binge drinking^b^ vs. Non-drinking^a^				Binge drinking^c^ vs. Non-drinking^a^			
Characteristics	PR	*p*-value^d^	aPR	*p*-value^e^	PR	*p*-value^e^	aPR	*p*-value^e^
**Age at interview (in years)**		**0.02**		**0.03**		0.16		0.56
18–29	1.34 (0.99-1.82)		1.45 (1.03-2.02)		1.23 (0.76-2.02)		0.92 (0.54-1.58)	
30–39	1.25 (1.01-1.53)		1.27 (1.03-1.57)		0.81 (0.50-1.33)		0.79 (0.51-1.21)	
40–44	Reference		Reference		Reference		Reference	
**Race/ethnicity** ^f^		0.87		0.60		0.32		0.29
Non-Hispanic black	Reference		Reference		Reference		Reference	
Hispanic or Latino^g^	0.93 (0.66-1.33)		0.99 (0.69-1.44)		1.19 (0.71-1.99)		1.39 (0.89-2.17)	
Non-Hispanic white	0.95 (0.69-1.30)		0.85 (0.61-1.18)		1.5 (0.85-2.64)		1.17 (0.71-1.94)	
**Highest educational attainment**		**<0.01**		**0.04**		0.51		
<High school	Reference		Reference		Reference			
High school graduate or GED	1.19 (0.88-1.61)		1.12 (0.83-1.51)		1.42 (0.66-3.02)			
>High school	1.56 (1.22-1.99)		1.39 (1.04-1.86)		1.02 (0.54-1.92)			
**Living at or below poverty level** ^h^		**<0.01**		**<0.01**		0.52		
No	1.71 (1.33-2.20)		1.47 (1.18-1.83)		1.19 (0.70-2.00)			
Yes	Reference		Reference		Reference			
**Health insurance coverage**		**<0.01**		0.20		0.19		
Any private insurance	1.51 (1.12-2.04)		1.28 (0.86-1.90)		1.12 (0.56-2.27)			
Public insurance only^i^	0.98 (0.74-1.31)		1.04 (0.75-1.44)		1.47 (0.92-2.34)			
Ryan White Coverage only or Uninsured	Reference		Reference		Reference			
**Current smoker**		**0.04**		0.06		**<0.01**		**<0.01**
No	Reference		Reference		Reference		Reference	
Yes	1.27 (1.01-1.60)		1.30 (1.00-1.68)		3.61 (2.38-5.47)		2.31 (1.38-3.87)	
**Any drug use** ^j^		**<0.01**		**0.03**		**<0.01**		**<0.01**
No	Reference		Reference		Reference		Reference	
Yes	1.47 (1.19-1.81)		1.31 (1.04-1.65)		4.05 (3.02-5.44)		2.25 (1.44-3.52)	
**Had condomless sex with a negative or unknown male partner**		**0.02**		0.40		**<0.01**		0.55
No	Reference		Reference		Reference		Reference	
Yes	1.36 (1.07-1.72)		1.11 (0.87-1.41)		1.91 (1.25-2.91)		1.16 (0.71-1.91)	
**Needed drug or alcohol counselling or treatment services** ^k^		**0.02**		**0.04**		**<0.01**		
No	1.66(1.03-2.68)		.73(0.97-3.09)		0.38(0.24-0.62)		0.73(0.39-1.36)	
Yes	Reference		Reference		Reference		Reference	
**ART adherent** ^l^		0.09		0.07		**<0.01**		**0.03**
No	1.27 (0.97-1.66)		1.27 (0.99-1.62)		2.36 (1.57-3.53)		1.56 (1.04-2.35)	
Yes	Reference		Reference		Reference		Reference	
**Virally suppressed** ^m^		0.09		0.72		**0.02**		0.11
No	1.18 (0.98-1.42)		1.05 (0.82-1.34)		1.55 (1.08-2.23)		1.40 (0.94-2.07)	
Yes	Reference		Reference		Reference		Reference	

Abbreviations: aPR = Adjusted Prevalence Ratio; ART = Antiretroviral Therapy; CI = Confidence interval; PR = Unadjusted Prevalence Ratio; GED = General Education Development **Bold** means significant at *p*<0.05.

Time period: In the past 12 months, unless otherwise noted. All measures are self-reported unless otherwise noted.

a Defined as women who did not report drinking any alcohol in the past 30 days.

b Defined as women who reported consuming ≥1 drink in the past 30 days and did not report consuming ≥4 drinks in a sitting.

c Defined as women who reported consuming ≥4 drinks during at least one sitting in the past 30 days.

d Percentages are weighted row percentages.

e Chi-square *p*-value is based on the Rao-Scott chi-square.

f Other racial groups are excluded because of small sample sizes.

g Hispanics or Latinos can be of any race.

h The number and percentage of participants meeting current poverty guidelines were determined using the U.S. Department of Health and Human Services poverty guidelines that corresponded to the calendar year for which the combined household income was asked.

i Public insurance includes Medicare, Medicare, Medicare or Medicaid with Ryan White, or any local or nationally funded health insurance.

j Drug use includes non-injection and injection use for non-medical reasons.

k Needed drug or alcohol counselling or treatment services is defined as patients who responded affirmatively that they needed drug or alcohol counseling or treatment services in the past 12 months. Among those who responded affirmatively that they needed the services (n=77), 66/77 (85.7%) responded that they needed and received drug or alcohol counselling or treatment services in the past 12 months; 11/77 (11.3%) responded that they needed and did not receive drug or alcohol counselling or treatment services in the past 12 months.

l ART adherent was defined as patients who self-report that they are currently taking ART and were 100% dose adherent in the past 3 days. A patient is defined as 100% adherent if they took their ART doses or set of pills/spoonfuls/injections of ART medications as prescribed by a health care provider in the last 3 days. Otherwise, they were considered as not adherent.

m Recent viral suppression is defined as the most recent viral load in the past 12 months prior to the interview as undetectable or <200 copies/ml. This information is based on data as recorded by medical record abstraction during the past 12 months prior to interview.
