# Supplementary material for: Alcohol use among HIV-positive women of childbearing age, United States, 2013–2014
Source: AIDS Care. Author manuscript; Available in PMC 2024 Apr 8. (PMC11000390; doi:10.1080/09540121.2020.1808161)
Supplement: Sup Table 2 [file NIHMS1976944-supplement-Sup_Table_2.docx]

**Appendix Table 2. Alcohol Consumption Measures among HIV-positive Women of Childbearing Age (18-44 years) in Care Who Drink, Overall and by Drinking Status ─ Medical Monitoring Project, 2013─2014**

| **Alcohol consumption measures** | **All drinkers** | | **Current drinkers/non-binge^a^** | | **Binge drinkers^b^** | |
| --- | --- | --- | --- | --- | --- | --- |
|  | **n** | **Weighted %** | **n** | **Weighted %** | **n** | **Weighted %** |
| Number of drinking days during past 30 days^c^ |  |  |  |  |  |  |
| 1-2 | 213 | 56.0 | 177 | 63.1 | 36 | 36.2 |
| 3-4 | 82 | 21.8 | 62 | 23.8 | 20 | 16.1 |
| ≥5 | 77 | 22.2 | 33 | 13.1 | 44 | 47.8 |
| Number of days consuming 4 or more drinks in one sitting during past 30 days^d^ |  |  |  |  |  |  |
| 1-2 | -^e^ | -^e^ | -^e^ | -^e^ | 59 | 55.4 |
| 3-4 | -^e^ | -^e^ | -^e^ | -^e^ | 23 | 21.9 |
| ≥5 | -^e^ | -^e^ | -^e^ | -^e^ | 18 | 22.7 |
| Total number of alcoholic drinks consumed during past 30 days^f^ |  |  |  |  |  |  |
| 1 | 57 | 15.2 | 56 | 20.0 | 1 | 1.1 |
| 2-3 | 93 | 24.9 | 90 | 32.2 | 3 | 3.2 |
| 4-5 | 56 | 14.5 | 44 | 15.7 | 12 | 11.3 |
| 6-8 | 56 | 16.1 | 41 | 16.1 | 15 | 16.1 |
| 9-11 | 19 | 5.7 | 9 | 4.1 | 10 | 10.7 |
| 12-14 | 11 | 3.5 | 8 | 3.7 | 3 | 3.0 |
| 15-20 | 26 | 6.2 | 12 | 4.0 | 14 | 12.6 |
| 21-30 | 7 | 1.5 | 2 | 0.5 | 5 | 4.2 |
| ≥31 | 42 | 12.4 | 9 | 3.7 | 33 | 38.0 |

^a^ Defined as women who reported consuming ≥1 drink in the past 30 days and did not report consuming ≥4 drinks in a sitting.

^b^ Defined as women who reported consuming ≥4 drinks during at least one sitting in the past 30 days.

^c^ Assessed by the following survey question: “During the past 30 days, on how many days did you have an alcoholic drink?”

^d^ Assessed by the following survey question: “During the past 30 days, on how many days did you have 4 or more alcoholic drinks in one sitting?”

^e^ Data applicable to women who reported binge drinking only.

^f^ Assessed by multiplying the number of drinking days during past 30 days by the number of alcoholic drinks consumed on a typical drinking day.
