# Supplementary material for: Alcohol use among HIV-positive women of childbearing age, United States, 2013–2014
Source: AIDS Care. Author manuscript; Available in PMC 2024 Apr 8. (PMC11000390; doi:10.1080/09540121.2020.1808161)
Supplement: Sup Table 1 [file NIHMS1976944-supplement-Sup_Table_1.docx]

**Appendix Table 1. Selected Questions in the Medical Monitoring Project Questionnaire, 2013 ─ 2014**

| **Reproductive History Questions**  Q1. Since testing positive for HIV in __ __/__ __ __ __ ***[INSERT DATE OF 1^st^ POSITIVE HIV TEST]***, how many times have you been pregnant? ***[PREG_9]***  ***Instructions to the interviewer:***  ***If number of times pregnant [PREG_9] is 1–5, SAY:*** “Now I would like to ask you about all of your pregnancies since testing positive for HIV, starting with your first one.”  ***If number of times pregnant [PREG_9] is greater than 5 pregnancies, SAY:*** “Now I would like to ask you about your first 5 pregnancies since testing positive for HIV.”  Q2. For your **1^st^** pregnancy since testing positive for HIV, were you trying to get pregnant? ***[INTNT1N3]***  No  0  Yes  1  Q3. What was the outcome of this pregnancy? ***[OUTCM1N3]***  Currently pregnant  1  ***Skip to Q5***  Live birth  2  Stillbirth  3  Miscarriage  4  Abortion  5  Q4. If not currently pregnant, in what month and year did this outcome occur? ***[PDTE1N3]***  __ __/ __ __ __ __  ^(M M / Y Y Y Y )^  Q5. If currently pregnant, what is your due date? ***[DUDT1N3]***  __ __/ __ __ __ __  ^(M M / Y Y Y Y )^  ***Repeat questions Q2-Q5 for pregnancy #2 – pregnancy #5.*** |
| --- |
| **Unmet Treatment and Counseling Needs Questions**  ***Instructions to the interviewer:***  ***SAY****:* I’m going to ask about services you used or needed during the **past 12 months**, (That is, from last year, (***DATE WITH PREVIOUS YEAR***) to now (***INTERVIEW DATE***).”  First I'll ask whether you received the service; then I'll ask whether you needed this service.  A1. During the **past 12 months**, did you get: Drug or alcohol counseling or treatment?  No  0  Yes  1  ***Instruction to Interviewer***: IF “No,” “Refused to answer,” or “Don’t know,” to Question A1 ASK:  A2. During the **past 12 months**, have you needed: Drug or alcohol counseling or treatment?  No  0  Yes  1 |
